# *Cryptococcus tetragattii* Meningitis Associated with Travel, Taiwan

**DOI:** 10.3201/eid2902.221425

**Published:** 2023-02

**Authors:** Pin-Han Wu, Chih-Hao Chen, Yu-Tzu Lin, Yu Ao, Kuo-Hsi Lin, Wen-Hsin Hsih, Chia-Huei Chou, Chih-Yu Chi, Mao-Wang Ho, Po-Ren Hsueh

**Affiliations:** China Medical University Hospital, Taichung, Taiwan (P.-H. Wu, C.-H. Chen, Y.-T. Lin, Y. Ao, W.-H. Hsih, C.-H. Chou, C.-Y. Chi, M.-W. Ho, P.-R. Hsueh);; Tungs' Taichung MetroHarbor Hospital, Taichung (K.-H. Lin)

**Keywords:** *Cryptococcus tetragattii*, meningitis/encephalitis, fungi, Africa, Taiwan, *Suggested citation for this article*: Wu P-H, Chen C-H, Lin Y-T, Ao Y, Lin K-H, Hsih W-H, et al. *Cryptococcus tetragattii* meningitis associated with travel, Taiwan. Emerg Infect Dis. 2023 Feb [*date cited*]. https://doi.org/10.3201/eid2902.221425

## Abstract

Meningitis caused by *Cryptococcus tetragattii* fungus is rare and has been found in specific geographic regions. We report a case of meningitis caused by *C. tetragattii* (molecular type VGIV) in an immunocompetent patient in Taiwan. The patient had traveled to Egypt and was positive for granulocyte-macrophage colony-stimulating factor autoantibody.

Meningitis caused by members of *Cryptococcus* *gattii* species complex is found in specific geographic or climatic regions (*1*). The incidence rate of *C.* *gattii*–caused meningitis is much lower than that caused by *C.* *neoformans *sensu stricto*.* We report a case of meningitis caused by *C. tetragattii* in an immunocompetent patient in Taiwan.

A 59-year-old female taxi driver with no systemic diseases sought care at China Medical University Hospital in Taichung, Taiwan, because she had experienced intermittent headache with unsteady gait for 2 months. She had received 1 dose of the ChAdOx1 nCoV-19 vaccine (AstraZeneca, https://www. astrazeneca.com) 4 months before admission. She had no contact history with pets, birds, or other animals. She frequently traveled abroad before the COVID-19 pandemic and had visited Italy, Egypt, and mainland China during 2018–2019. 

After she was admitted, brain magnetic resonance imaging revealed mild cortical swelling with narrowing of the cerebral sulci in the bilateral medial frontoparietal lobes, indicating meningitis or leptomeningeal carcinomatosis. We performed lumbar puncture and obtained cerebrospinal fluid (CSF) on the second hospitalization day. Studies on the CSF were positive for yeasts by India ink staining and showed a cryptococcal antigen titer of 1:40× (CrAg LFA; IMMY, https://www.immy.com). We detected *C. neoformans* and *C. gattii* by using a multiplex PCR panel for meningitis (BioFire ME Panel; bioMérieux, https://www.biomerieux.com). Fungal culture of the CSF specimen yielded *Cryptococcus* species. We identified the organism as *C. gattii*; its score value was 1.92 by Bruker Biotyper matrix-assisted laser desorption/ionization time-of-flight mass spectrometry system (Bruker Daltonics GmbH, https://www.bruker.com). We performed multilocus sequence typing (MLST) and phylogenetic analysis MLST for 7 genetic loci, including *CAP59*, *GPD1*, *IGS1, LAC1*, *PLB1*, *SOD1*, and the *URA5* region, as previously described (*2*), and detected *C. gattii* ST576. We deposited sequence data of the 7 foci from MLST into GenBank (accession nos. OP828681–87). The URA5 gene restriction fragment length polymorphism of the *C. gattii* isolate performed as previously described (*3*); the results showed that this isolate belonged to the VGIV genotype, *C. tetragattii* (CMU-3028) (*2*).

A serial survey that included autoimmune profiles (antinuclear antibody, rheumatoid factor, anti-neutrophil cytoplasmic antibody, anti–double stranded DNA, complement component 3, and complement component 4), HIV-1/2 antibodies, and free HIV-1 p24 antigen (HIV-1/2 Ag/Ab Comb test; Abbott Laboratories, https://www.abbott.com) all showed negative results, but the result for the anti-granulocyte-macrophage colony-stimulating factor autoantibody (anti-GM-CSF AAb) was positive (titer 0.84), as measured using ELISA as previously described (*4*).

Induction treatment for the cryptococcal meningitis involved liposomal amphotericin B (200 mg/d) plus oral flucytosine (1,250 mg/6 h) for 21 days. A follow-up CSF culture revealed negative growth on hospital day 15. Oral voriconazole (250 mg/12 h) was prescribed as consolidation treatment for 15 days. The patient was discharged in stable condition and followed up at the outpatient department. She received oral fluconazole (400 mg/d) for the subsequent 6 months and remained well.

In this case, the patient is an immunocompetent host who had previously received 1 dose of the ChAdOx1-S vaccine and had a travel history to Egypt 2 years before symptom onset of meningitis. The members of the *C. gattii* species complex has different geographic distribution, and *C. tetragattii* is mostly isolated in Africa (*5*). Approximately 20% of the HIV-infected tested population had cryptococcal meningitis caused by *C. tetragattii* in Zimbabwe (*6*). In addition, anti-GM-CSF AAb has been detected in immunocompetent patients with *C. gattii* infection, as well as those with *C. neoformans* infection (*4*).

Whether COVID-19 vaccines have a trigger effect in unmasking underlying diseases, such as that in our patient, warrants further study. To our knowledge, no studies demonstrate the relationship between anti-GM-CSF AAb and COVID-19 vaccines. However, research showed that autoantibody such as anti–smooth muscle antibody was triggered by COVID-19 vaccine as autoimmune hepatitis (*7*). In conclusion, clinicians should be aware of possible *C. tetragattii* fungal infection in patients who have traveled to meningitis-endemic regions.
